# Development of an LC–MS/MS Method for Simultaneous Quantification of Erythromycin A and Seven Metabolites: Initial Residue Profile in Salmon Muscle with Skin

**DOI:** 10.3390/molecules31193406

**Published:** 2026-09-24

**Authors:** Chaitali Chattopadhaya, Sean Knutson, Hernando Escobar Loaiza, Kenneth M. Taylor, Charles Gieseker, Pak-Sin Chu

**Affiliations:** Division of Residue Chemistry, Office of Applied Science, Center for Veterinary Medicine, U.S. Food and Drug Administration (FDA), Laurel, MD 20708, USAsean.knutson@fda.hhs.gov (S.K.); charles.gieseker@fda.hhs.gov (C.G.);

**Keywords:** veterinary drug residues, antibiotics, LC–MS/MS, erythromycin A, erythromycin metabolites, analytical quantitation, Atlantic salmon

## Abstract

A robust liquid chromatography tandem mass spectrometry (LC–MS/MS) method was developed and validated for determining erythromycin A (ERY A) and its metabolites: erythromycin B (ERY B), erythromycin C (ERY C), erythromycin A N-oxide (ENO), anhydroerythromycin A (AHEA), N-demethylerythromycin A (NDEA), erythromycin A enol ether (EAEE), and pseudoerythromycin A enol ether (PSEE) in salmon muscle with skin. Tissue samples (1.00 g) fortified with ERY A, ERY B, ERY C, ENO, EAEE, and PSEE at 50–200 ng/g and AHEA and NDEA at 500–2000 ng/g, using a surrogate internal standard, were extracted with acetonitrile, defatted hexane, and diluted water, resulting in accuracy from 87% to 108%, precision from 3% to 13%, and estimated LOQ from 0.02 to 1.2 ng/g. Incurred salmon showed ERY A as the major residue at 3 days post-administration and NDEA and AHEA as potential markers exceeding the 500 ng/g level on Day 28.

## 1. Introduction

Antibiotics are used in the aquaculture industry to manage bacterial infections of farmed aquatic animals. However, the indiscriminate use of antibiotics in fish grown for food is a significant concern, especially among developing nations with limited regulatory oversight [1]. These fish may contain excess antibiotic residues due to fish farmers using unapproved compounds or improperly dosing approved formulations without following proper withdrawal times before placing them into commerce. The consumption of fish tissue with excess antibiotic residues can increase health hazards regarding antimicrobial resistance [2]. To mitigate human exposure to drug residues, regulatory agencies in the European Union, Japan, and the United States conduct human food safety assessments of new animal drugs used in animals intended for consumption based on scientific data [3]. Studies such as these that generate data for safety assessments require chemical methods that can detect the full profile of a drug and its metabolites so that a marker residue can be determined. The marker residue can be the parent drug or metabolites whose concentrations have a correlation with the level of total residues in a specific edible tissue.

Atlantic salmon (*Salmo salar*) aquaculture has expanded exponentially in the last 40–50 years, spreading across many countries in the Northern and Southern Hemispheres [4]. In addition, a variety of other salmonid species are also extensively grown for food (e.g., rainbow trout, *Oncorhynchus mykiss*) and for the enhancement of wild stocks. Bacterial kidney disease (BKD), a major disease problem of salmonid fishes cultured in fresh and saltwater environments, is a slow-growing chronic disease caused by the Gram-positive bacterium, *Renibacterium salmoninarum* [5]. In the United States, two formulations of the macrolide antibiotic erythromycin are under new investigational animal drug applications [6,7]. The goal is to determine if the antibiotic can effectively control mortality caused by BKD in a variety of salmonid species and prevent the vertical transmission of *R. salmoninarum* between salmonid broodstock. The primary active ingredient of the formulations under investigation is erythromycin A (ERY A), CAS No. 114-07-8, as shown in Figure 1, which has antibacterial activity against Gram-positive bacteria [8]. To establish the safety of drug residues and to control contamination in food products, the FDA must identify an appropriate marker residue of erythromycin so it can set a tolerance, which is the maximum concentration of the marker residue that can remain in a specific edible tissue of a treated animal [9].

Currently, a semi-quantitative microbiological assay is used for the approval and investigational use of erythromycin in salmonids by regulatory agencies. This method has set the tolerance of ERY A as 100 ng/g based on antimicrobial activity [9]. However, the microbiological method is non-specific, labor-intensive, and time-consuming. No studies show the total residue and metabolism data that could suggest an appropriate marker residue for erythromycin in salmon. For this reason, a more sophisticated chemical detection method is preferred for monitoring erythromycin residues in salmonid food fishes, ensuring food safety and consumer protection.

After administration, ERY A undergoes enzymatic biotransformation reactions in the liver that form metabolites. The identification and characterization of potential metabolites is necessary to determine the safety, efficacy, and toxicity of the drug. Traditionally, radiolabeled drugs have been used to interpret absorption, distribution, and metabolism of a drug in vitro and in vivo [10,11]. The technique does not provide structural information of the drug or its mass spectrometry ionization properties. Modern LC–MS/MS methods are very useful for accurate and precise determination of the parent drug as well as metabolites in the target tissue of the test system using reference standards.

During the last decade, various multiclass and multiresidue methods have been reported for the screening and quantification of ERY A in fish tissue among a mixture of various compounds using LC–MS/MS techniques [12,13,14,15], which have provided reductions in the cost and time of drug analysis in edible fish. Additionally, several chromatographic methods have been published in the literature for the analysis of the parent drug ERY A only, without its metabolites, in fish [16,17,18,19]. One of these methods requires an additional detector for confirmatory analysis due to the lack of structurally useful fragment ions necessary for the validation of chemical identity [16]. Some methods are slow, labor-intensive, and use complicated sample pre-treatment processes [17,18]. The extraction procedure may involve complex parameter adjustments and may lack specificity and stability under extreme conditions [19]. To our knowledge, this is the first validated LC–MS/MS method enabling simultaneous detection and structural confirmation of ERY A and its metabolites, employing a surrogate internal standard alongside certified reference standards for each individual metabolite.

Radiolabeled studies have been a powerful tool for metabolite identification in animal tissues [10,11]. Once metabolites are identified, a suitable marker is selected for the development of a regulatory method. For ERY A, this approach is not feasible because its synthesis is difficult and costly. As an alternative, we have explored an approach of identifying possible metabolites by searching against commercially available reference standards. Upon the successful identification of the potential metabolites, we further verify their presence or absence in tissues obtained from dosed fish. The present study describes the development and validation of an LC–MS/MS method for the determination and identification of ERY A and seven metabolites (ERY B, ERY C, ENO, EAEE, PSEE, NDEA, and AHEA) in Atlantic salmon muscle with skin using erythromycin-(N-methyl-^13^C, d_3_) as the internal standard and certified reference standards for metabolites. Our objectives are to determine the metabolite profile of ERY A in salmon and to identify potential marker residues for monitoring purposes. The method assumes a presumptive tolerance of 100 ng/g in salmon based on the official microbiological method.

## 2. Results and Discussions

### 2.1. Method Development and Validation

Initially, liquid–liquid extraction using acetonitrile followed by evaporating the extract to dryness under a stream of nitrogen at 55 °C was used. The crude extract was defatted with hexane. In addition, this initial method had extra steps such as the sonication and filtration of the final extract through a syringe filter before LC–MS/MS analysis. A matrix-matched calibration curve was used for quantitation. The samples were analyzed using an atmospheric pressure chemical ionization mass spectrometry (APCI–MS) technique. The laboratory results were not reproducible. Later, the extraction method was simplified further by omitting the steps of evaporation, sonication, and filtration through a syringe filter. The updated method eliminated the use of a matrix-matched calibration curve, and quantification was performed by a solvent calibration curve using electrospray ionization mass spectrometry (ESI–MS). In this method, the homogenized samples (ground frozen with liquid nitrogen using a food processor) were subjected to liquid–liquid extraction twice using acetonitrile. Supernatants were collected and combined. Only 1 mL of the crude extract was defatted using hexane. The clean extract was diluted 1:1 with water, centrifuged, and analyzed by the LC–MS/MS system.

The extraction efficiency of the method was within ±10% as measured by the quotient of the area ratio of the Quan transition of the analyte and ISERY in the pre-fortified control muscle extract and the area ratio of the analyte and ISERY in the post-fortified control muscle extract. The ME was within ±10% for all analytes, indicating a less significant matrix effect.

The reversed-phase C18 columns from Agilent and Phenomenex were tested at the beginning relying on their hydrophobicity, but good separations of all the metabolites could not be achieved by using water containing 0.1% formic acid and acetonitrile as the mobile phase. Later, chromatographic separation was optimized by using the Phenomenex Kinetex Biphenyl uHPLC column (100 Å, LC Column 100 × 2.1 mm) supported by its selectivity properties, which primarily rely on a mixed-mode approach: π–π interactions, as the biphenyl ligand contains aromatic rings that strongly interact with any conjugated systems or electron-rich areas in the erythromycin structure, and dipole–dipole interactions where the phase is effective at interacting with polar functional groups, such as the desosamine and cladinose sugar moieties, and hydroxyl groups present on the erythromycin macrocyclic ring.

Different gradient programs were tested with mobile phases consisting of water containing 0.1% formic acid and acetonitrile. The biphenyl column yielded symmetrical peak shapes, provided better separation of interfering peaks for the analytes, and gave reproducible retention times for all compounds determined in this method.

The mass spectrometer was optimized for each analyte using a concentrated standard solution (1 µg/mL) dissolved in methanol by syringe infusion. The operating parameters for the MS instrument—TEM, CUR, GS 1 and 2, CAD, and V voltage—were automatically adjusted and optimized using Sciex Analyst software (Version 1.7.2). The protonated molecules were selected as precursor ions for multiple reaction monitoring (MRM) transitions from the precursor ions to the product ions in positive ion mode. Collision energy (CE) and other variable voltage parameters such as collision cell exit potential (CXP) were optimized for each analyte (Table 1). Declustering potential (DP) and entrance potential (EP) presented stable signals for all ions with values predominantly of 11 and 10, respectively.

Figure 2A–C shows representative MRM chromatograms for mixed standards in a solvent, control muscle fortified with mixed standards, and an incurred sample 14 days post-incursion with erythromycin A. In all cases, the RTs are consistent for all compounds irrespective of the matrix. No major interfering peaks were detected in negative control salmon muscles at the RT window of the analytes. Although small co-eluting peaks were observed in some of the control extracts because of the high sensitivity of the instrument Sciex 6500 QTRAP (AB Sciex, Foster City, CA, USA), the concentrations were significantly less than the lowest calibration point (25 ng/g or 250 ng/g). The assay of sample preparation combined with chromatography was free from major interfering components at the RT of the analytes.

The reproducibility of the chromatographic system was evaluated in each batch by injecting a 100 ng/g mixed standard five times at the beginning of each batch. The average value from three batches indicated a CV% for RT of <1% and a CV% for accuracy of <10% for all analytes.

Calibration curve linearity was good for all analytes in two sets of curves run on matrices from three sources. The corresponding correlation coefficient (r) was >0.995 for all analytes using 1/x weighting.

The validation experiment of fortified muscle samples from three different sources indicated that the accuracy for all analytes was between 87% and 108%, within the limits of 60–115% (for 10 ng/g to <100 ng/g fortification levels) or 80–110% (for ≥100 ng/g fortification levels), as stated in FDA guidance. The CV% was between 2.9% and 12.8%, within the limits of ≤22% (for 10 ng/g to <100 ng/g fortification levels) or ≤11% (for ≥100 ng/g fortification levels) for all other analytes except AHEA and ERY B at the 200 ng/g fortification level (Table 2). The estimated LOQ for all analytes ranged from 0.02 to 1.2 ng/g, determined for each analyte with the following values: 0.8 ng/g ERY A; 0.2 ng/g ERY B; 0.07 ng/g ERY C; 0.02 ng/g ENO; 0.2 ng/g EAEE; 0.02 ng/g PSEE; 1.2 ng/g AHEA, and 0.8 ng/g NDE.

The confirmation criteria listed in the guidance for industry were applied [20,21]. The false-positive rate and the false-negative rate successfully met the recommendations [20].

The diluted incurred salmon sample from day 14 at two different ratios (Table 3) indicated average concentrations for ERY A, NDEA, and AHEA. For the 1:8 ratio sample, the concentrations were 103 ng/g, 592 ng/g, and 650 ng/g, respectively. For the 1:4 ratio sample, the concentrations were 183 ng/g, 1086 ng/g, and 1103.5 ng/g, respectively. The CV% for concentrations of ERY A, NDEA, and AHEA for the 1:8 ratio sample were 5%, 4%, and 5.5%, and for the 1:4 ratio sample, they were 10.5%, 8%, and 10%, respectively.

### 2.2. Metabolic Profile of Erythromycin A and Potential Metabolites in Atlantic Salmon Muscle

After successful validation of the quantitative method, an analysis of freshly generated incurred muscles indicated that the parent compound ERY A and the metabolites AHEA and NDEA were the major residues in post-dose muscle samples collected on day 14 (Figure 2C). ERY A residue was dominant in the day 3 sample, which decreased rapidly thereafter and decreased to less than the lower limit used for the method validation of 50 ng/g (Figure 3). The post-administration sample period revealed that the residue level of AHEA initially increased before decreasing with time. In the day 28 post-dose sample, the highest level of AHEA was in the edible muscle, followed by NDEA and ERY A (Figure 3, Table 4). The residue levels of the other analytes were relatively low in comparison to ERY A, NDEA, and AHEA during the days of the post-dose sample.

## 3. Materials and Methods

### 3.1. Chemical Standards and Reagents

Analytical reference standards were acquired from BioAustralis (NSW 2164, Australia) with a purity >95%. Solid reference standards of ERY A (BIA-E1311), ERY B (BIA-E1350), ERY C (BIA-E1351), NDEA (BIA-D1352), AHEA (BIA-A1348), ENO (BIA-E1539), EAEE (BIA-E1347), and PSEE (BIA-P1349) were pre-weighed by the manufacturer and supplied in 5 mL amber glass vials (Table 5 and Figure 1) and shipped with a certificate of analysis. Methanol (MeOH), acetonitrile (ACN), and formic acid (A117-10XAMP) were all of Optima LC–MS grade (Fisher Scientific, Pittsburgh, PA, USA). Hexanes were of HPLC grade and obtained from Fisher Scientific. Ultrapure water was generated in-house by a Millipore water purification system (Milli-Q IQ 7000, EMD Millipore, Burlington, MA, USA). The internal standard (ISERY), erythromycin-(N-methyl-^13^C, d_3_) of 98% purity (product number 663506), was purchased from Sigma-Aldrich (St. Louis, MO, USA). For salmon tissue incursion, an investigational medicated feed premix containing erythromycin thiocyanate (100 g/lb.; CAS No. 7704-67-8) in wheat flour (ADM Animal Nutrition, Quincy, IL, USA) was acquired from the investigational new drug application sponsor, AquaTactics, Inc. (Kirkland, WA, USA).

### 3.2. Preparation of Stock, Intermediate Solutions, Calibration Standard Solutions, and Quality Control Samples

#### 3.2.1. Stock Standard Solutions

Individual pre-weighed reference standards, supplied by the manufacturer in 5 mL vials, were dissolved each in 4 mL MeOH. They were stored at ≤−15 °C for ≤6 months.

#### 3.2.2. Intermediate Standard Solution (2 µg/mL and 20 µg/mL)

Stock standard solution was vortexed, sonicated, and transferred quantitively using calibrated micropipettes (Eppendorf, CT USA) to a 10 mL volumetric flask to make a mixed intermediate standard of 2 µg/mL of ERY A, ERY B, ERY C, ENO, EAEE, and PSEE and 20 µg/mL of NDEA and AHEA, respectively. Intermediate standard solution of analytes was stored at ≤−15 °C for ≤6 months.

#### 3.2.3. Calibration Standards Solution

The mixed calibration standard solutions of ERY A, ERY B, ERY C, ENO, EAEE, and PSEE at concentrations of 1, 2.5, 5, 10, 20, and 40 ng/mL and NDEA and AHEA at concentrations of 10, 25, 50, 100, 200, and 400 ng/mL were prepared in 20:80 (*v*/*v*) acetonitrile/water by dissolving appropriate volumes of mixed intermediate stock standard (2 or 20 µg/mL) in glass centrifuge tubes.

For ERY A, ERY B, ERY C, ENO, EAEE, and PSEE, the solvent standards were equivalent to 25, 50, 100, 200, and 400 ng/g, and for NDEA and AHEA, standards were equivalent to 250, 500, 1000, 2000, and 4000 ng/g in a 1 g salmon muscle sample. A higher calibration curve for NDEA and AHEA was prepared because the residues from incurred fish exceeded 500 ng/g after 28 days of dosing the fish. The freshly prepared calibration standards were stored at ≤−15 °C for ≤3 months.

#### 3.2.4. Internal Standard Stock and Intermediate Solution

A 10 µg/mL internal standard stock solution of ISERY was prepared in MeOH by accurately weighing 1 ± 0.1 mg of neat standard in a 100 mL volumetric flask using an analytical balance (Mettler Toledo XPE105, Columbus, OH USA). A 2 µg/mL intermediate internal standard solution of ISERY was prepared in MeOH by diluting 5 mL of the stock solution in a 25 mL volumetric flask. The internal standard stock solution was stored at ≤−15 °C for ≤12 months in darkness. The intermediate internal standard solution was stored at ≤−15 °C for ≤6 months.

#### 3.2.5. Quality Control Salmon Samples

Positive control salmon muscle samples were prepared by fortification of muscle tissues using the mixed intermediate standard solution containing 2 µg/mL of ERY A, ERY B, ERY C, ENO, EAEE, and PSEE; 20 µg/mL of NDEA and AHEA; and 2 µg/mL of intermediate internal standard solution of ISERY.

### 3.3. LC–MS/MS Instrument

The analysis was performed using a Nexera-X2 Ultra-High-Performance Liquid Chromatography (UHPLC) system from Shimadzu Scientific Instruments (Columbia, MD, USA), which consisted of three pumps (LC–30AD), an autosampler (SIL-30AC), and a column compartment (CTO-20AC). Chromatographic separation was achieved using a Kinetex^®^ 2.6 µm Biphenyl 100 Å 100 × 2.1 mm column (Phenomenex, Torrance, CA, USA) protected with Security Guard ULTRA Cartridges for UHPLC Biphenyl 2.1 mm ID column.

Mobile phases consisted of 0.1% formic acid in water and 100% acetonitrile. A gradient with a flow rate of 0.3 mL/min consisted of increasing acetonitrile from 25% at 0 min to 40% at 8 min and then 100% from 10 to 12 min, followed by decreasing to 25% from 12.1 to 15 min. Column and autosampler temperatures were maintained at 40 °C and 10 °C, respectively. Injection volume was 2 μL.

The UHPLC system was connected to a Sciex 6500 mass spectrometer. The detector had a TurboIon Spray source, which was operated in positive electrospray ionization mode. The optimized analytical parameters were source temperature (TEM), 550 °C; curtain gas (CUR), 23 psi; ion source gas 1 (GS1), 80 psi; ion source gas 2 (GS2), 60 psi; collision gas (CAD), medium; and ion spray voltage (V), 5500 V.

The acquisition was performed in multiple reaction monitoring (MRM) mode. Identification and quantification of compounds were performed with Sciex Analyst (1.7.2) software. Mass spectrometer parameters and ion transitions are listed in Table 5. For each analyte, three MS–MS transitions (one quantification ion and two qualifier ions) were acquired and used for confirmatory analysis. Ion chromatogram peaks were first identified using Analyst software (Version 1.7.2) with preset parameters.

Signal-to-noise ratio (S/N), retention time (RT), ion abundance ratio, and area threshold were used to confirm the analytes in each sample. Ion ratios were calculated from the relative abundance of each qualifier ion to its corresponding quantification ion. Ion ratios should be ±20% of the average of the standards. The S:N ratios should be greater than 3:1 for confirmatory ions in samples, and the RT repeatability should be ≤5% [20,21,22].

### 3.4. Salmon Muscle Tissue Samples

All fillet samples were salmon muscle with intact skin. Control salmon fillets with skin were purchased from local grocery stores and used for the method validation experiments. Control and incurred muscle tissue samples were generated for the incursion experiments. Atlantic salmon (*Salmo salar*) were obtained from the U.S. Department of Agriculture, National Cold Water Marine Aquaculture Center (Franklin, ME, USA) as juveniles and grown to market size in recirculating freshwater aquaculture systems at 12 ± 2 °C. The fish were fed commercial fish feed and were not administered any drug or chemical treatments prior to this study. Two Atlantic salmon (547 and 832 g, respectively) were not dosed and sacrificed to generate control fillet tissue. To administer treatments, the fish were held in a tank supplied with a constant flow of the same water used for the holding systems at the same water temperature. Six Atlantic salmon (750 ± 147 g) were administered with the investigational medicated feed premix at the rate of 100 mg/kg body weight in a gelatin capsule via oral gavage administration for three consecutive days (1 fish/timepoint). The dosed fish were removed and sacrificed on days 3, 7, 10, 14, 21, and 28 post-drug administration. Two fillet samples were collected and stored frozen in an ultra-low temperature freezer at <−70 °C.

Before grinding, the samples were thawed at room temperature in deionized water for 3–4 h, dried with paper towels, and chopped into small pieces. The pieces were then chilled with liquid nitrogen and ground in a 6 L industrial food processor (Robot Coupe, Jackson, MS, USA) for 30 s. Grounded samples were divided into smaller portions in labeled polypropylene cups. Samples were weighed on the same day (1.00 ± 0.07 g) in 50 mL conical polypropylene (PP) centrifuge tubes (Corning, NY, USA) and stored at <−70 °C in a freezer until extraction.

For method verification purpose, the incurred salmon muscle from day 14 was diluted to two different ratios, 1:8 and 1:4, by mixing thoroughly with ground control salmon muscle samples and stored at <−70 °C in a freezer.

### 3.5. Sample Extraction

Pre-weighed samples were removed from the ultra-low-temperature freezer and thawed at room temperature for 1–1.5 h before processing. The positive quality control samples were fortified at 50, 100, and 200 ng/g, respectively, for ERY A, ERY B, ERY C, ENO, EAEE, and PSEE and at 500, 1000 and 2000 ng/g for NDEA and AHEA using the intermediate stock standard mix and intermediate internal standard of ISERY. The tubes were vortexed briefly (Vortex-mix-Genie 2 mixer, Scientific Industries, Inc., Bohemia, NY, USA), and then three to four borosilicate glass beads 4 mm in size (Kimble Chase, 13500-4, Fisher, Pittsburgh, PA, USA) and 2 mL of acetonitrile were added in each tube. All samples were agitated in a multitube vortexer VWR VX-2500 (VWR Scientific Products, Buffalo Grove, IL, USA) for 5 min, then centrifuged (Sorvall Legend XFR programmable refrigerated benchtop centrifuge set at 4 °C, with TX-750 swinging bucket rotor) at 3148× *g* for 5 min. The acetonitrile layer was transferred to a 15 mL conical PP centrifuge tube. The remaining tissue was re-extracted with an additional 2 mL of acetonitrile, and the supernatant was collected in the same 15 mL tube and combined with the previously collected extract. The final volume of the extract was adjusted to 5 mL with acetonitrile and vortex mixed. An amount of 1 mL of the crude extract was washed with 2 mL hexane to remove fat. The hexane phase was discarded. The clean extract in ACN was diluted 50:50 (*v*/*v*) with Milli-Q water in a 2 mL PP microfuge tube (Eppendorf, CT, USA), vortexed, and centrifuged at 16,000× *g* (Spectrafuge 24D by Labnet, Edison, NJ, USA) for 10 min. The supernatant was transferred to a 2 mL glass autosampler vial and 2 µL was injected into the LC–MS/MS system.

### 3.6. Method Validation

Method validation was performed following FDA guidelines [21,23]. Untreated samples of Atlantic salmon muscle from three different sources were used for method validation. Each batch consisted of a duplicate negative control, a negative control fortified with internal standard, and control muscles fortified with intermediate mixed standard at three different levels (between 50 and 200 ng/g for ERY A, ERY B, ERY C, ENO, EAEE, and PSEE and between 500 and 2000 ng/g for NDEA and AHEA). Incurred salmon muscle from day 14 was diluted to two ratios, 1:8 and 1:4. Each sample was run in duplicate on three different days.

Prior to sample analysis, system suitability testing was performed. A calibration standard was injected and evaluated against the following performance criteria. The product ions of different analytes with the highest signal ion count (“Quan” transition) of five replicates of the calibration standard (100 ng/g/1000 ng/g) should have a signal-to-noise ratio ≥10. The coefficient of variation (CV%) of analyte peak area should be ≤5%, and the RT repeatability for the eight analytes and ISERY should be ≤5%.

Data from each analyte was processed independently. The quantitative method was evaluated based on the performance parameters of selectivity, limit of detection, limit of quantitation, linearity, range, accuracy, precision, and spike accuracy, as indicated in FDA guidance [21]. The acceptability of the entire dataset in each batch includes system suitability injections, calibrators, quality control samples, and incurred muscle samples.

Linear calibration curves were constructed by plotting the peak area of each analyte against corresponding nominal concentrations in the calibrators. To accept a run, each calibration curve should have a correlation coefficient (r) ≥ 0.990. The back-calculated accuracy of the calibration points of an analyte should be within ± 10% of the nominal value. In each batch, two sets of calibration curves were generated, one at the beginning and one at the end, bracketing the sample injection sequence to compensate for variation of the instrument response with time.

Precision and accuracy of the method were estimated by measuring concentrations of each analyte in quality control (QC) samples on three different days. Precision was reported as CV% of the measured values at each QC level, and accuracy was expressed as relative error percentage between the mean measured value and the nominal value at that level. The calculated accuracy for fortified samples should be between 60 and 115% for fortification levels of 10 ng/g to <100 ng/g and between 80 and 110% for fortification levels ≥ 100 ng/g with coefficients of variation (CV%) of ≤10% [21].

Matrix effect (ME) was evaluated by comparing the peak area ratio of the Quan-transition for the analyte and ISERY in post-extraction fortified control extracts with those acquired in solvent standard solutions using the formula

(X/Y − 1) × 100%

where

X = the area ratio of analyte and ISERY in solvent standard;

Y = the area ratio of analyte and ISERY in post fortified control muscle extract.

The sample extraction procedure and chromatographic conditions were optimized to minimize ion suppression/enhancement, with an acceptance criterion of ±10% for matrix effects. Table 6 presents matrix effect values calculated for salmon samples purchased from three retail locations. All results met the established acceptance criterion.

Method accuracy of the analytes was determined based on two calibration curves measured using the same set of standards. The linear concentration range was established by running the solvent standards at five levels ranging from 25 ng/g to 400 ng/g for ERY A, *ERY* B, ERY C, ENO, EAEE, and PSEE and from 250 ng/g to 4000 ng/g for NDEA and AHEA, once at the beginning and once at the end, bracketing the sample injection sequence. Accuracy of QC samples was calculated by comparing the concentrations determined by the calibration curve with corresponding theoretical concentrations. Assuming acceptable precision, the average method accuracy range should be 60–115% and 80–115% depending on whether the designated concentration of the proposed marker residue is less than 100 ng/g or ≥ 100 ng/g, respectively [21].

The selectivity of the method was evaluated by comparing the chromatograms obtained from matrix blanks and matrix spikes for three different matrices used during validation. The selectivity of the LC–MS/MS method was assessed to confirm that no matrix-derived isobaric interferences, co-eluting species, or in-source fragments contributed meaningfully to the signal at the precursor-to-product ion transitions monitored for the target analyte and internal standard. It was evaluated by analyzing unspiked matrix blanks alongside matrix-spiked samples prepared for each of the three matrices employed during validation and salmon incurred samples. All samples were analyzed using the multiple reaction monitoring (MRM) transitions optimized for each analyte with a *m*/*z* [precursor] → *m*/*z* [product ion 1] (quantifier) and two *m*/*z* [precursor] → *m*/*z* [product ion 2,3] (qualifiers), as well as the corresponding transitions for the internal standard (*m*/*z* [IS precursor] → *m*/*z* [IS product ion]).

In all blank matrix extracts, no interfering peaks were observed at the chromatographic retention time of the analytes. Similarly, no interferences of the internal standard response were detected in the transition channel. These acceptance criteria are consistent with FDA guidance [24]. The inherent selectivity of the triple quadrupole mass spectrometer—afforded by the dual mass filtering of precursor and product ions in the SRM acquisition mode—provided high discrimination against non-specific matrix components. This selectivity was further supported by consistent ion ratios (quantifier-to-qualifier transition ratios) between matrix-spiked samples, incurred salmon samples, and neat calibration standards, with observed ratios below ±20% of the reference value, confirming the absence of co-eluting isobaric interferences that could bias quantification (Supplementary Tables S1 and S2). The Supplementary Materials show how selectivity was demonstrated by overlaying MRM chromatograms of blank matrix, blank fortified with mixed standards, and incurred samples (Supplementary Figures S1–S10). No interfering peaks were observed at analyte retention times.

The LOQ was estimated according to FDA guidance [25] using the criterion of S/N ≥ 10 and verified by acceptable accuracy and precision at the LOQ level. The reported LOQ values were experimentally confirmed using stepwise dilution of spiked blank salmon muscle.

### 3.7. Samples Stability

For method implementation, an analyst requires information about the stability of sample extracts when a re-assay is needed due to instrument malfunction or unforeseen circumstances. In this method development, we re-assayed the same sample extracts over several time points, simulating our experimental conditions and comparing analytical results. Extract and standard preparation stability was performed on different days, calculating deviation from the original value, and taking as the criteria for acceptability results of ≤15% [26]. In addition, for ERY A stability in incurred salmon samples, a previous report indicates that ERY A is stable at room temperature [23] and degrades in fish to NDEA and AHEA only after high-temperature treatment [27]. Therefore, storing the incurred salmon and profile study samples at <−70 °C until extraction, preservation of any ERY A incurred and derived metabolites was assured throughout this profile evaluation.

### 3.8. Research Approach Limitations

The residue profile study utilized one fish per timepoint and took two samples from each fish. While this design provided an initial characterization of tissue residue profiles, it may not fully capture inter-individual biological variability, which can influence the rate and extent of a metabolite depletion. Consequently, the statistical robustness is inherently limited. Future studies employing larger sample sizes would improve the precision and generalizability of metabolite depletion kinetics and support a more comprehensive assessment of withdrawal interval recommendations.

## 4. Conclusions

The results demonstrate that this straightforward, validated, and sensitive LC–MS/MS method is effective for the quantitative analysis of ERY A and seven potential metabolites in Atlantic salmon muscle with skin using commercially available reference standards. Isotopically labeled ERY A as an internal standard demonstrates that the method has high precision and accuracy for all tested analytes. ERY A, NDEA, and AHEA are the major residues in the salmon muscle samples collected at different time points after administration of the drug. The metabolite profiles indicated that AHEA and NDEA remain as the major residues after a withdrawal period of 28 days, suggesting that these metabolites can be potential marker residues for monitoring erythromycin in salmon.

## Figures and Tables

**Figure 1:**
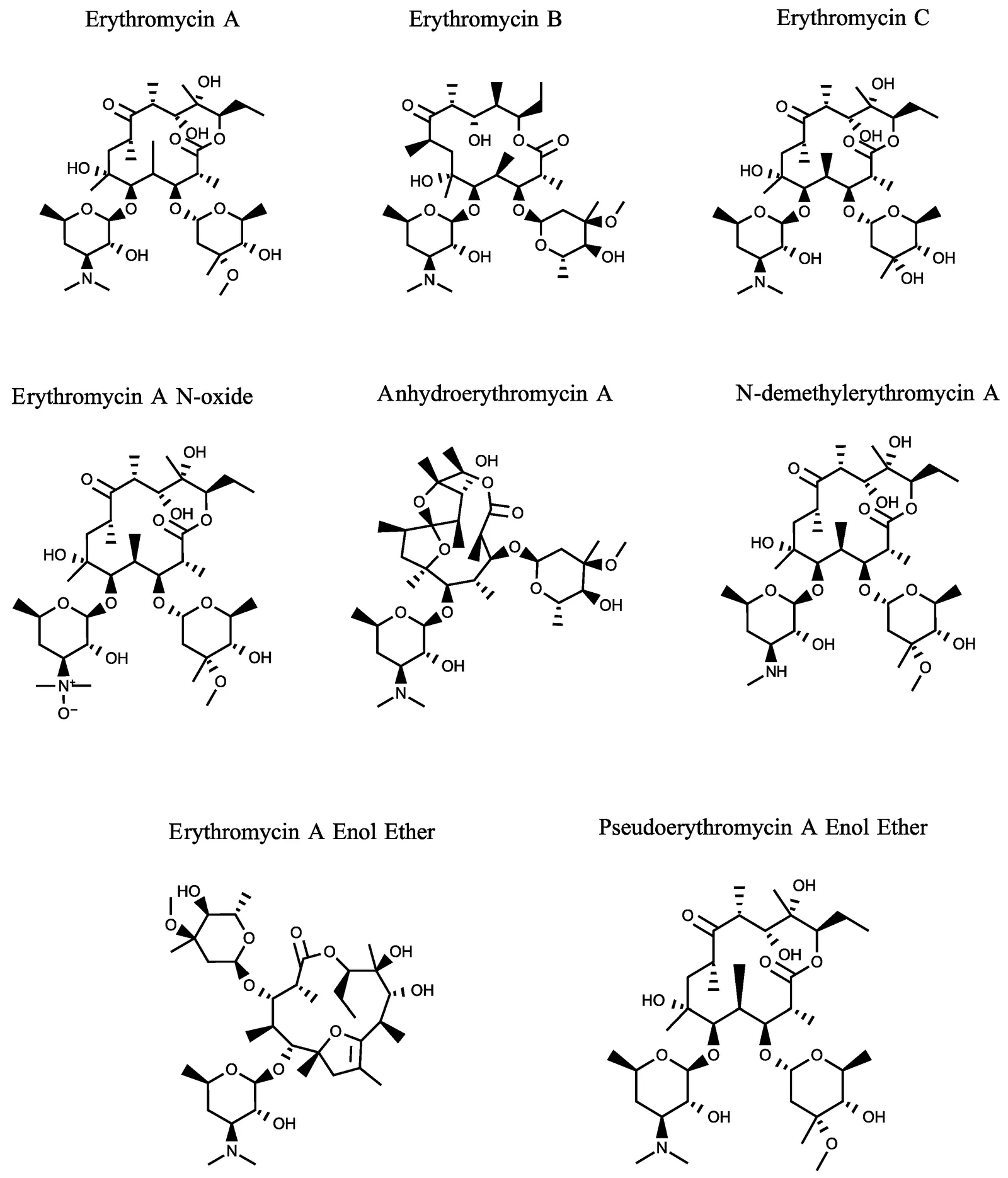
Chemical structures for erythromycin A (ERY A) and metabolites included in the study: erythromycin B (ERY B); erythromycin C (ERY C); erythromycin A N-oxide (ENO); anhydro erythromycin A (AHEA); N-demethylerythromycin A (NDEA); erythromycin A enol ether (EAEE); and pseudo erythromycin A enol ether (PSEE). Structures were drawn using ChemDoodle (iChemLabs, Version 12.7.0).

**Figure 2:**
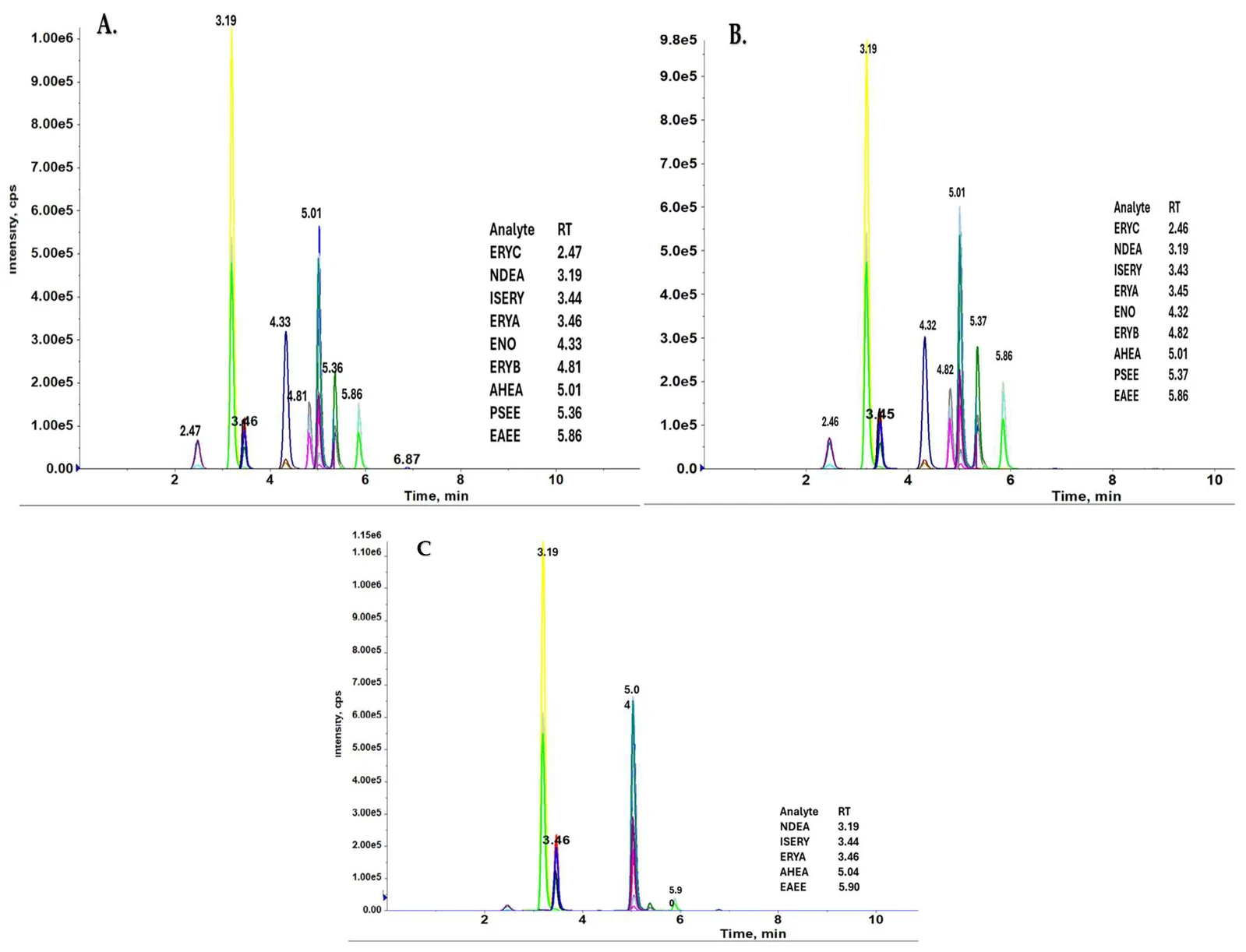
Representative MRM chromatograms for standards in solvent and incurred salmon muscle: (**A**) mixed standards in solvent at 50 ng/g, (**B**) a control salmon muscle sample fortified with mixed standards at 50 ng/g, and (**C**) an incurred salmon muscle sample at day 14 post-dose of erythromycin thiocyanate at 100 mg/kg for three consecutive days.

**Figure 3:**
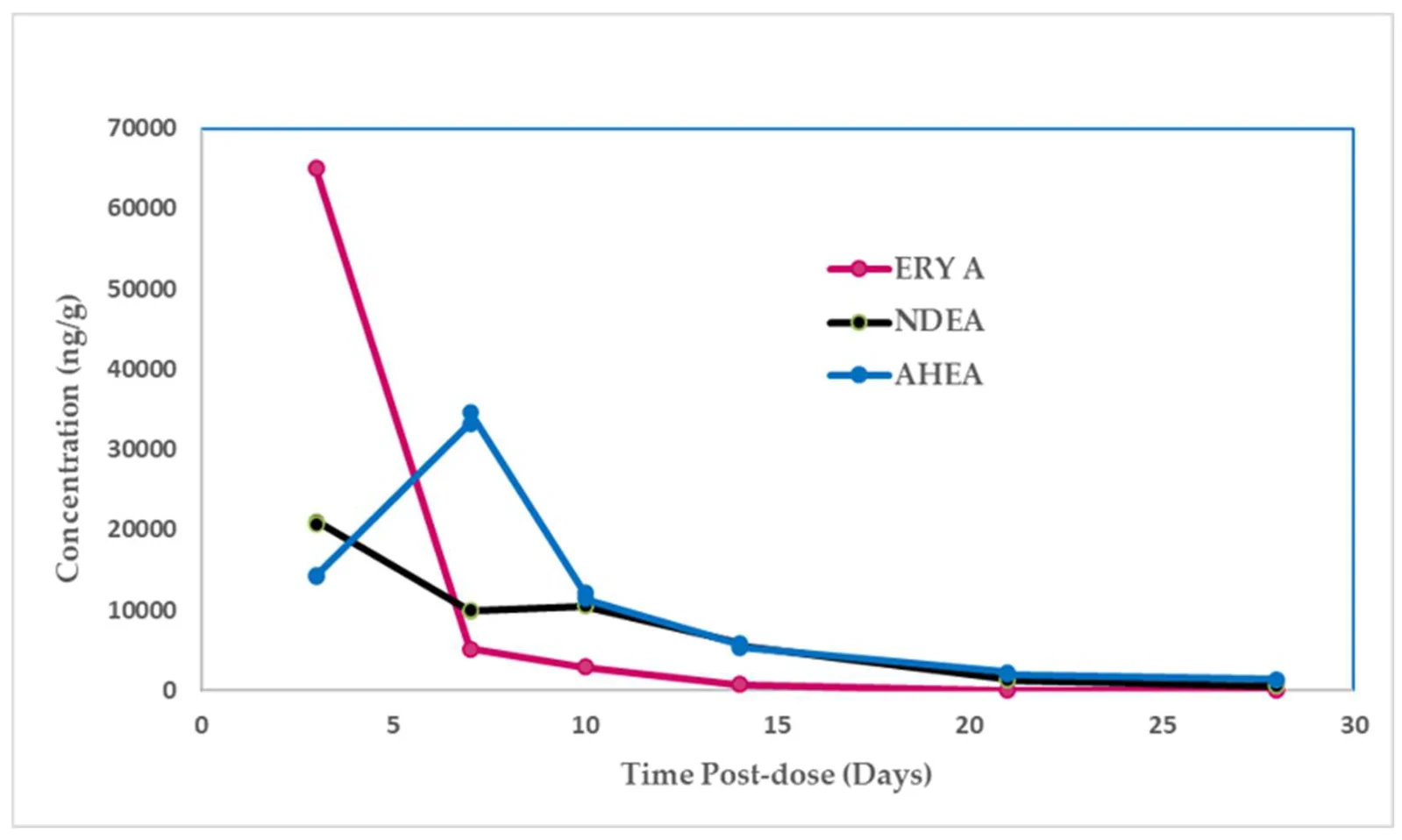
Residue profile level of ERY A and dominant metabolites NDEA and AHEA in incurred Atlantic salmon muscle with skin after the withdrawal period.

**Table 1:** Precursor-product ion transitions and compound-dependent mass spectrometer parameters identified during mass spectrometry optimization. Quantitation ions per analyte are highlighted in bold.

ID	Q1 Mass *m*/*z*	Q3 Mass *m*/*z*	CE	CXP
**ERY A**	**734.5**	**158.0**	**37**	**18**
	734.5	576.2	27	14
	734.5	83.0	83	10
**ENO**	**750.4**	**592.2**	**33**	**14**
	750.4	574.2	33	14
	750.4	123.0	51	8
**ERY B**	**718.5**	**158.1**	**35**	**14**
	718.5	560.4	25	14
	718.5	542.2	27	14
**ERY C**	**720.5**	**158.0**	**35**	**18**
	720.5	576.3	25	14
	720.5	558.4	33	10
**NDEA**	**720.5**	**144.1**	**33**	**10**
	720.5	562.2	27	14
	720.5	82.9	91	10
**AHEA**	**716.4**	**158.1**	**35**	**10**
	716.4	540.3	27	12
	716.4	82.9	83	12
**EAEE**	**716.4**	**158.1**	**35**	**10**
	716.4	558.4	21	14
	716.4	540.3	27	12
**PSEE**	**716.4**	**158.1**	**35**	**10**
	716.4	558.4	21	14
	716.4	82.9	83	12
**ISERY**	**738.5**	**580.3**	**23**	**26**

**Table 2:** Average accuracy and precision (CV%) for all analytes in different fortification levels.

Analyte	Fort. Level 50 ng/g		Fort. Level 100 ng/g		Fort. Level 200 ng/g	
	Accuracy %	CV%	Accuracy %	CV%	Accuracy %	CV%
**ERY A**	102.5	3.0	100.0	6.5	101.0	3.0
**ERY C**	95.0	4.0	94.0	8.0	97.0	6.0
**NDEA**	90.0	4.0	93.0	5.0	97.0	4.0
**ENO**	87.0	9.0	90.0	6.0	96.0	8.0
**EAEE**	105.0	5.0	101.5	7.0	106.0	8.0
**AHEA**	102.0	8.0	101.0	4.0	101.0	11.0
**PSEE**	103.5	8.0	98.0	6.5	101.0	8.0
**ERY B**	104.5	6.0	99.0	8.0	108.0	13.0

**Table 3:** Inter-day precision and accuracy for concentration of analytes in fortified control and incurred salmon muscle sample from day 14 post-administration. Six replicates per assay.

Analyte	Fortification Level (ng/g)	Mean (ng/g)	SD	CV%	Accuracy %
**ERY A**	50	51.0	2.0	3.0	102.5
	100	100.0	6.5	6.5	100.0
	200	202.5	6.0	3.0	101.0
	Incurred sample L1(Day14_1:8 dilution)	103.0	5.0	5.0	N/A
	Incurred sample L2(Day14_1:4 dilution)	183.0	19.0	10.5	N/A
**NDEA**	500	452.0	16.0	4.0	90.0
	1000	926.0	50.0	5.0	93.0
	2000	1934.0	70.0	4.0	97.0
	Incurred sample L1(Day14_1:8 dilution)	592.0	22.0	4.0	N/A
	Incurred sample L2(Day14_1:4 dilution)	1086.0	86.0	8.0	N/A
**AHEA**	500	510.0	42.0	8.0	102.0
	1000	1009.0	44.5	4.0	101.0
	2000	2015.0	224.0	11.0	101.0
	Incurred sample L1(Day14_1:8 dilution)	650.0	36.0	5.5	N/A
	Incurred sample L2(Day14_1:4 dilution)	1103.5	108.5	10.0	N/A

**Table 4:** ERY A and metabolite concentrations (ng/g) in Atlantic salmon muscle at different day timepoints following erythromycin thiocyanate administration (100 mg/kg) ª.

		ERY A	NDEA	AHEA	EAEE	ERY C	PSEE	ERY B	ENO
Days post administration	3	66,480.0	28,320.0	22,480.0	8120.0	1688.0	507.1	272.0	9.1
	7	6675.0	9695.0	37,800.0	748.5	323.5	75.6	15.3	5.0
	10	3680.0	10,135.0	12,000.0	651.5	286.0	75.6	4.7	4.5
	14	806.4	5032.0	4908.0	87.2	81.0	40.6	1.7	1.0
	21	33.2	1385.0	2075.0	6.7	6.8	3.8	<LOQ	<LOQ
	28	16.1	605.0	1420.0	5.9	3.3	2.1	0.3	<LOQ

ª Average value (*n* = 2).

**Table 5:** Diverse characteristics for erythromycin A and analogs used in this study.

Compound Name	Formula	CAS No.	Mol Wt.
**Erythromycin A (ERY A)**	C_37_H_67_NO_13_	114-07-8	733.9
**Erythromycin B (ERY B)**	C_37_H_67_NO_12_	527-75-3	717.9
**Erythromycin C (ERY C)**	C_36_H_65_NO_13_	1675-02-1	719.9
**Anhydroerythromycin A (AHEA)**	C_37_H_65_NO_12_	23893-13-2	715.9
**N-Demethylerythromycin A (NDEA)**	C_36_H_65_NO_13_	992-62-1	719.9
**Erythromycin A N-oxide (ENO)**	C_37_H_67_NO_14_	992-65-4	749.9
**Erythromycin A enol ether (EAEE)**	C_37_H_65_NO_12_	33396-29-1	715.9
**Pseudoerythromycin A enol ether (PSEE)**	C_37_H_65_NO_12_	105882-69-7	715.9
**Erythromycin-(N-methyl-13C, d3) (ISERY)**	^13^CC_36_d_3_H_64_NO_13_	2378755-05-9	737.9

**Table 6:** Assessment of matrix effects in control salmon muscle obtained from three grocery stores (n = 2). All results fell within the acceptable range of ±10%.

ID	Store 1	Store 2	Store 3
ERYA 1	0.9	1.0	3.4
ERYC 1	−2.9	−8.6	−5.4
NDEA 1	−1.8	−2.2	−0.9
ENO 1	−1.7	−3.5	−2.7
EAEE 1	6.2	5.6	6.1
AHEA 1	3.9	2.7	0.7
PSEE 1	6.8	3.7	4.8
ERYB 1	2.1	1.5	1.5

## Data Availability

The original contributions presented in this study are included in the article. Further inquiries can be directed to the corresponding author.

## Supplementary Materials

The following supporting information can be downloaded at: https://www.mdpi.com/article/10.3390/molecules31193406/s1, Figure S1. Summary of all MRM transitions for analyte compounds; Figure S2. Erythromycin A. MRM chromatograms; Figure S3. Erythromycin B. MRM chromatograms; Figure S4. Erythromycin C. MRM chromatograms; Figure S5. N-demethylerythromycin A. NDEA. MRM chromatograms; Figure S6. Anhydroerythromycin A. AHEA. MRM chromatograms; Figure S7. Erythromycin A N-oxide. ENO. MRM chromatograms; Figure S8. Erythromycin A enol ether. EAEE. MRM chromatograms; Figure S9. Pseudoerythromycin A enol ether. PSEE. MRM chromatograms; Figure S10. Internal standard (ISERY), erythromycin-(N-methyl-^13^C, d_3_) MRM chromatogra; Table S1. Ion ratio calculations determined ERY A, NDEA and AHEA; Table S2. Detailed calculations for ion ratio calculations determined on ERYA.

## Abbreviations

The following abbreviations are used in this manuscript:

UHPLC	Ultra-high-performance liquid chromatography
LC–MS/MS	Liquid chromatography tandem mass spectrometry
ERY A	Erythromycin A
ERY B	Erythromycin B
ERY C	Erythromycin C
ENO	Erythromycin A N-oxide
AHEA	Anhydro erythromycin A
NDEA	N-demethylerythromycin A
EAEE	Erythromycin A enol ether
PSEE	Pseudo erythromycin A enol ether
PP	Polypropylene
