# Supplementary material for: Development of an LC–MS/MS Method for Simultaneous Quantification of Erythromycin A and Seven Metabolites: Initial Residue Profile in Salmon Muscle with Skin
Source: Molecules. 2026 Sep 24;31(19):3406. doi: 10.3390/molecules31193406 (PMC13666175; doi:10.3390/molecules31193406)
Supplement: Supplementary file 1 [file molecules-31-03406-s001.zip › molecules-4497829-supplementary.pdf]

Supporting Information

Development of an LC-MS/MS Method for Simultaneous Quantitation of Erythromycin A and Seven Metabolites: Initial Residue Profile Study in Salmon Muscle with Skin

Chaitali Chattopadhaya, Sean Knutson , Hernando Escobar Loaiza *, Kenneth M. Taylor † ,
Charles Gieseker and Pak-Sin Chu †

Division of Residue Chemistry, Office of Applied Science, Center for Veterinary Medicine, U.S. Food and Drug Administration (FDA), Laurel, MD 20708, USA; sean.knutson@fda.hhs.gov (S.K.); charles.gieseker@fda.hhs.gov (C.G.)

* Correspondence: hernando.escobarloaiza@fda.hhs.gov

† Author retired.

Table of contents

1. MRM transitions for Erythromycin A and metabolites. ……….…………… S-2

Figure S1. Summary of all MRM transitions for analyte compounds………………… S-2

Figure S2. Erythromycin A. MRM chromatograms …………………………………….. S-3

Figure S3. Erythromycin B. MRM chromatograms…………………………………...... S-3

Figure S4. Erythromycin C. MRM chromatograms …………………………………… S-4

Figure S5. N-demethylerythromycin A. NDEA. MRM chromatograms .……………… S-4

Figure S6. Anhydroerythromycin A. AHEA. MRM chromatograms …………………… S-5

Figure S7. Erythromycin A N-oxide. ENO. MRM chromatograms…………………….. S-5

Figure S8. Erythromycin A enol ether. EAEE. MRM chromatograms……………….. S-6

Figure S9. Pseudoerythromycin A enol ether. PSEE. MRM chromatograms………….. S-6

Figure S10. Internal standard (ISERY), erythromycin-(N-methyl-^{13}C, d_3). MRM chromatograms……………………………………………………………………………… S-7

2. Ion ratio calculations for ERY A, NDEA, and AHEA — analytes present at high levels — throughout the 14-day post-dose sampling period………………………………………… S-7

Table S1. Ion ratio calculations determined on ERY A, NDEA and AHEA………………… S-7

Table S2. Detailed calculations for ion ratio calculations determined on ERYA… …….. S-8

1. **MRM transitions for Erythromycin A and metabolites.**
   This is the sequence found in all figures from S1 to S10: A. Blank salmon matrix, B. Salmon matrix fortified with standards at 50 ppb, C. Incurred salmon muscle sample at day 14 post-dose of erythromycin thiocyanate at 100 mg/kg.

   Note: non-relevant information fields were de-identified in some figures from the submitted copies.

Figure S1. Summary of all MRM transitions for analyte compounds.

A.

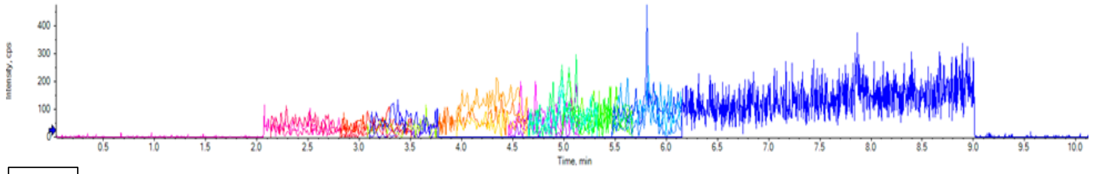

B.

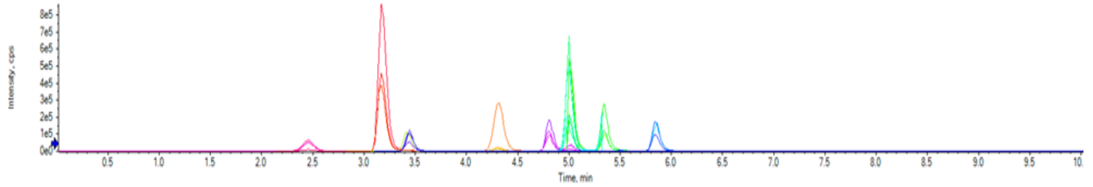

C.

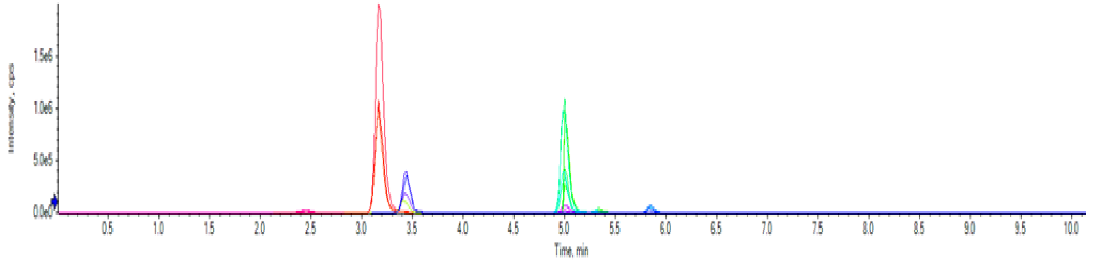

Figure S2. Erythromycin A. MRM chromatograms

ERY A

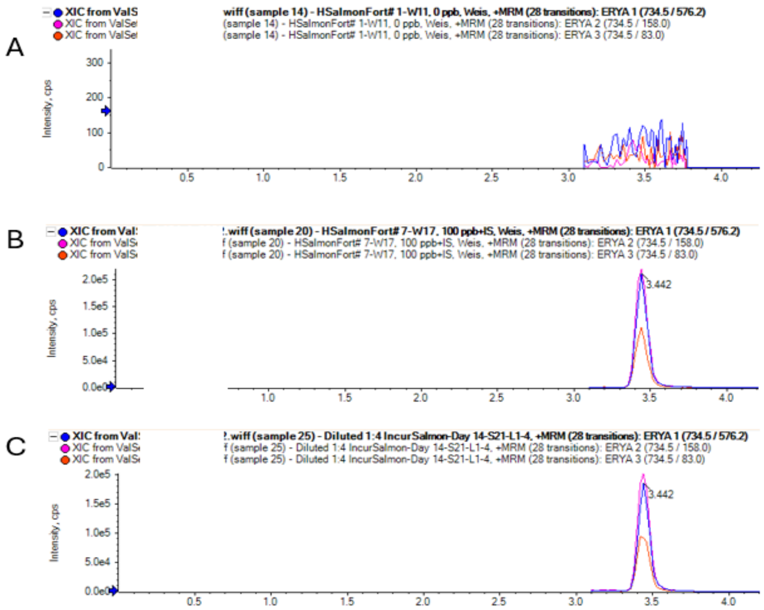

Figure S3. Erythromycin B. MRM chromatograms

ERY B

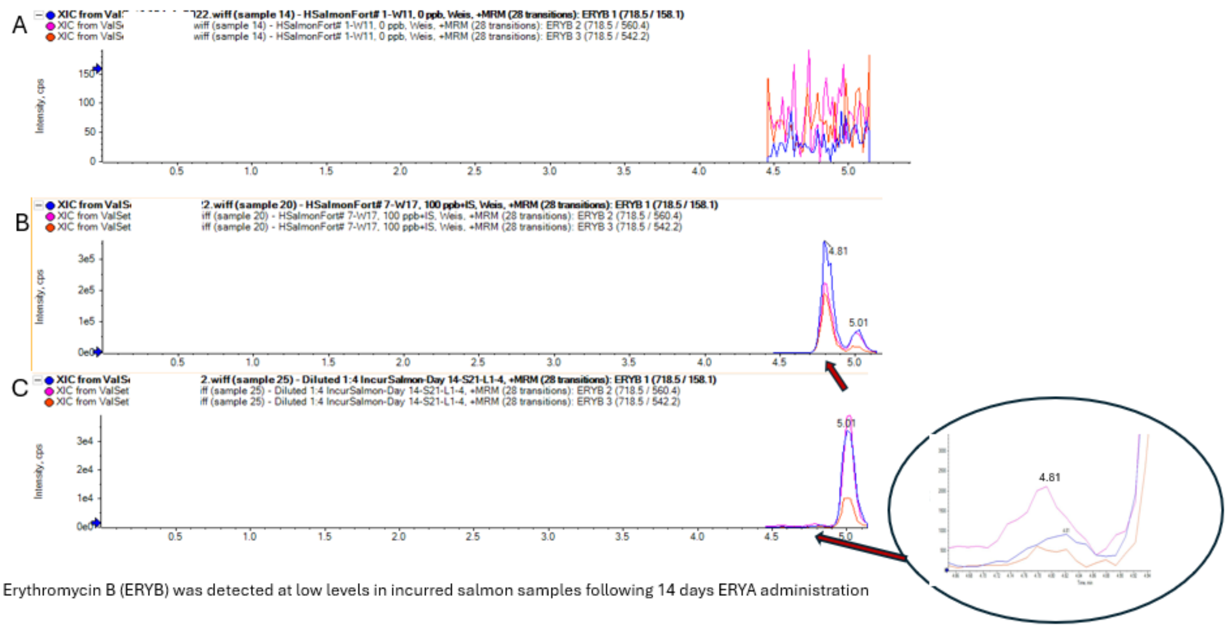

Erythromycin B (ERYB) was detected at low levels in incurred salmon samples following 14 days ERYA administration

Figure S4. Erythromycin C. MRM chromatograms

Ery C

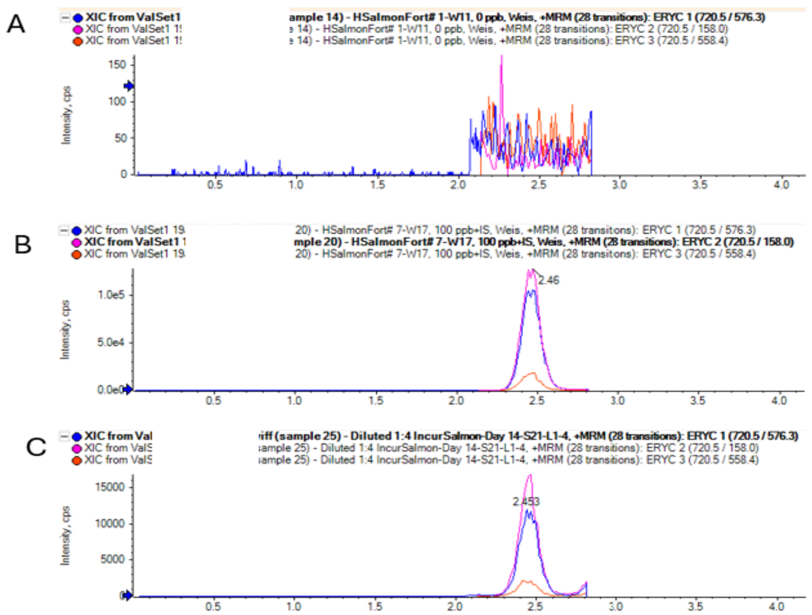

Figure S5. N-demethylerythromycin A. NDEA. MRM chromatograms

NDEA

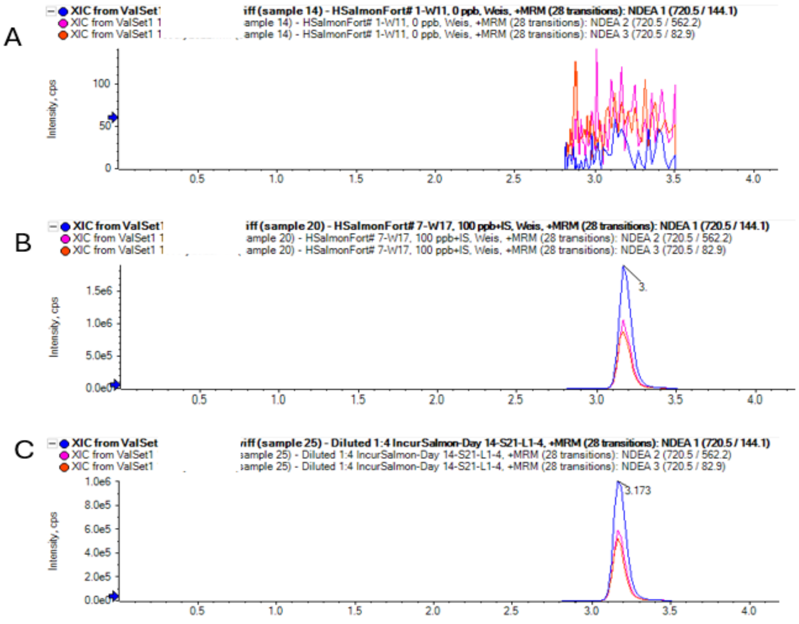

Figure S6. Anhydroerythromycin A. AHEA. MRM chromatograms

AHEA

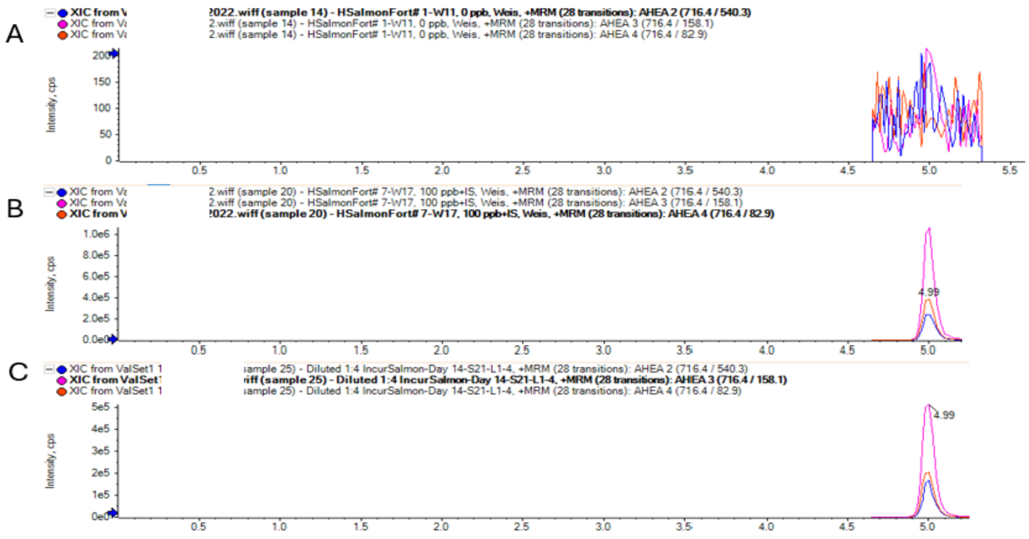

Figure S7. Erythromycin A N-oxide. ENO. MRM chromatograms

ENO

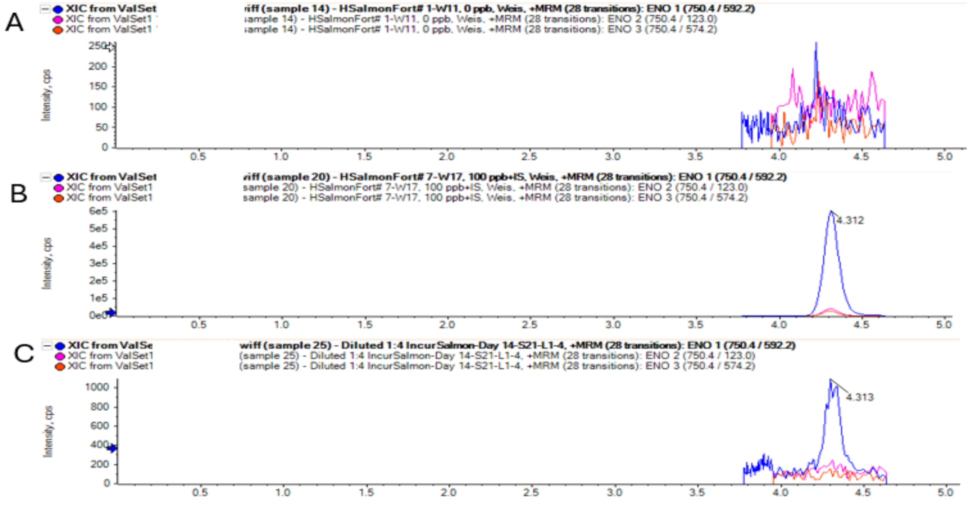

Figure S8. Erythromycin A enol ether. EAEE. MRM chromatograms

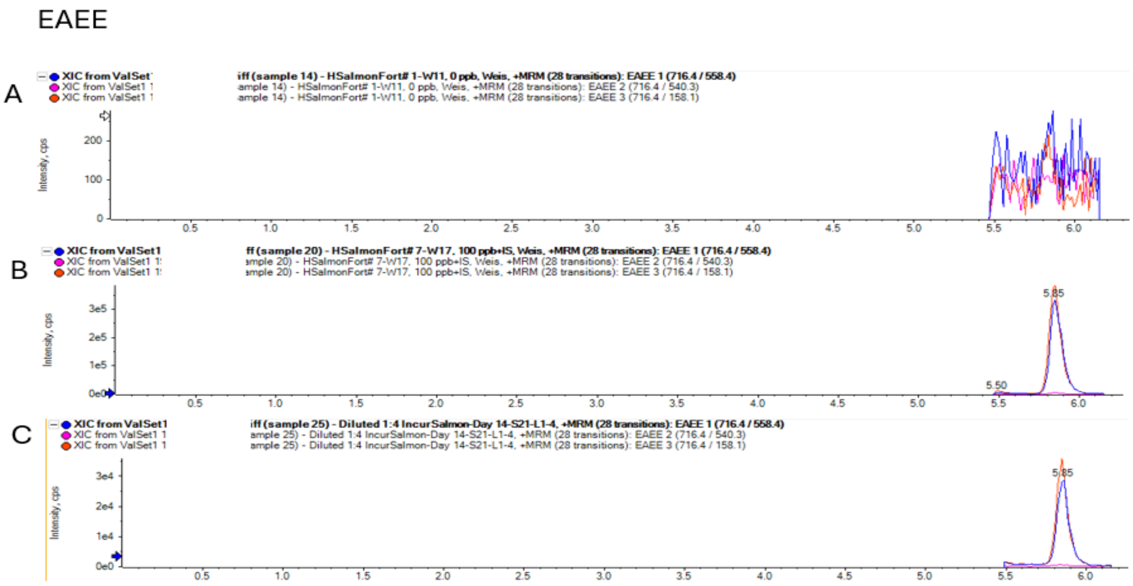

Figure S9. Pseudoerythromycin A enol ether. PSEE. MRM chromatograms

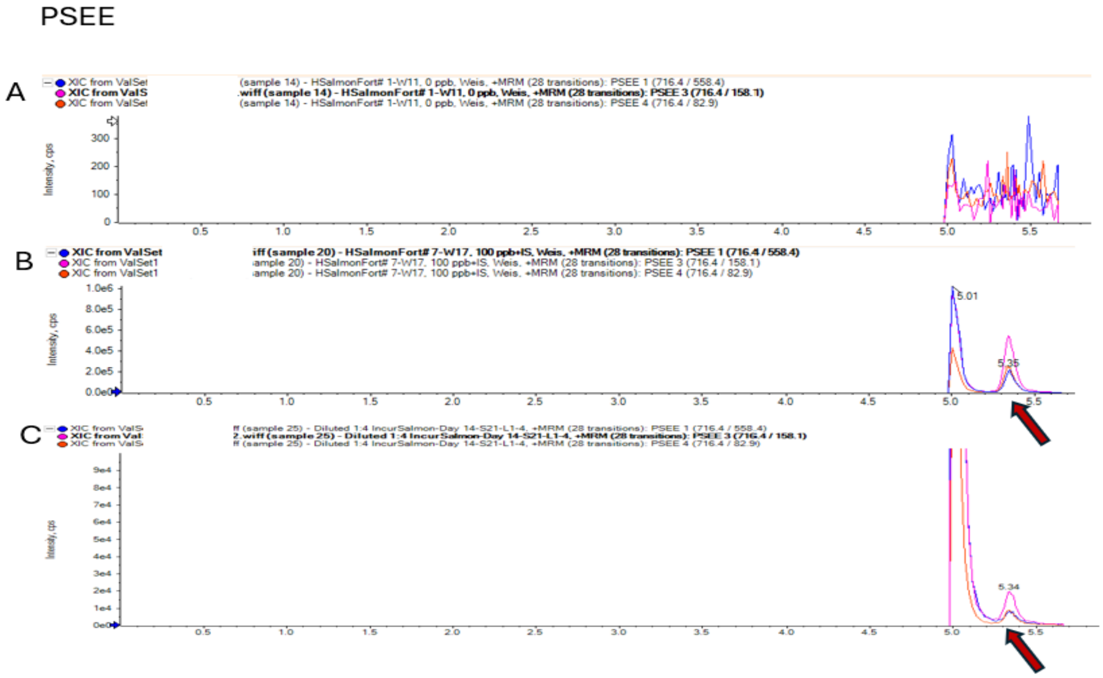

Figure S10. Internal standard (ISERY), erythromycin-(N-methyl-^{13}C, d_3). MRM chromatograms

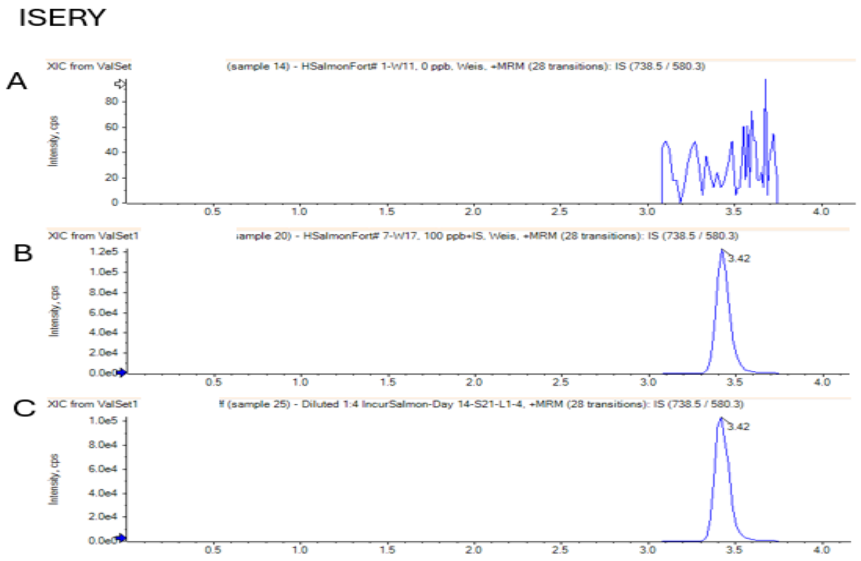

2. Ion ratio calculations for ERY A, NDEA, and AHEA — analytes present at high levels — throughout the 14-day post-dose sampling period.

Table S1. Ion ratio calculations determined on ERY A, NDEA and AHEA

	Difference ion ratio 1 with average standards, %			Difference ion ratio 2 with average standards, %		
**Sample**	**ERYA 1**	**AHEA1**	**NDEA1**	**ERYA 2**	**AHEA2**	**NDEA2**
Incur-Salmon-diluted 1:4-Day 14	-2.25	8.3	-2.0	-6.4	-3.1	-0.9
Incur-Salmon-diluted 1:4-Day 14-replicate	2.37	15.7	5.1	0.1	-2.2	3.6
Incur-Salmon-diluted 1:8-Day 14	5.26	17.5	-0.9	-0.5	-6.0	-1.5
Incur-Salmon-diluted 1:8-Day 14-replicate	2.81	16.8	-2.6	-2.4	-1.8	0.6

Table S2. Detailed calculations for ion ratio calculations determined on ERYA

Sample Name	Sample Type	Area ERYA Quantifier	Area ERYA Qualifier 1	Area ERYA Qualifier 2	Ion ration 1 Qual1/Quant %	Difference ion ratio 1 with Average standards %	Ion ratio 2 Qual2/Quant %	Difference ion ratio 2 with Average standards %
Solvent	Blank	ND	ND	ND	N/A		N/A	
Standard #1, 25 ppb	Standard	281800	228400	135300	81.05		48.01	
Standard#2, 50 ppb	Standard	599300	515800	300300	86.07		50.11	
Standard#3, 100 ppb	Standard	1173000	959200	572300	81.77		48.79	
Standard #4, 200 ppb	Standard	2314000	1813000	1133000	78.35		48.96	
Standard #5, 400 ppb	Standard	4610000	3549000	2344000	76.98		50.85	
Solvent	Blank	ND	ND	ND	N/A	N/A	N/A	N/A
Salmon Fortified #1-0 ppb	Quality Control	ND	ND	ND	N/A	N/A	N/A	N/A
Salmon Fortified #2- 0 ppb	Quality Control	ND	ND	ND	N/A	N/A	N/A	N/A
Salmon Fortified #3- 50 ppb	Quality Control	602200	527900	306700	87.66	6.97	50.93	3.30
Salmon Fortified #4- 50 ppb	Quality Control	631700	537600	305700	85.10	3.85	48.39	-1.85
Salmon Fortified #5- 100 ppb	Quality Control	1224000	1072000	592100	87.58	6.87	48.37	-1.88
Salmon Fortified #6- 100 ppb	Quality Control	1211000	1042000	605400	86.04	5.00	49.99	1.40
Salmon Fortified #7- 200 ppb	Quality Control	2241000	1733000	1084000	77.33	-5.63	48.37	-1.89
Salmon Fortified #8- 200 ppb	Quality Control	2352000	2078000	1166000	88.35	7.81	49.57	0.55
**Incur-Salmon-diluted 1:4-Day 14**	**Unknown**	**1220000**	**977300**	**563000**	**80.11**	**-2.25**	**46.15**	**-6.40**
**Incur-Salmon-diluted 1:4-Day 14-replicat**	**Unknown**	**1115000**	**935400**	**550100**	**83.89**	**2.37**	**49.34**	**0.07**
**Incur-Salmon-diluted 1:8-Day 14**	**Unknown**	**2271000**	**1959000**	**1114000**	**86.26**	**5.26**	**49.05**	**-0.51**
**Incur-Salmon-diluted 1:8-Day 14-replicat**	**Unknown**	**2121000**	**1787000**	**1021000**	**84.25**	**2.81**	**48.14**	**-2.36**
Solvent	Blank	N/A	N/A	N/A	N/A		N/A	
Standard #1, 25 ppb-2nd	Standard	288100	224100	139000	77.79		48.25	
Standard#2, 50 ppb-2nd	Standard	517100	440400	254800	85.17		49.27	
Standard#3, 100 ppb-2nd	Standard	1138000	1027000	586600	90.25		51.55	
Standard #4, 200 ppb-2nd	Standard	2392000	1915000	1189000	80.06		49.71	
Standard #5, 400 ppb-2nd	Standard	4622000	3790000	2197000	82.00		47.53	
				**Average standards**	**81.95**		**49.30**	

The relative abundance of the confirmation ions must be within ±20% of the absolute average of the standards.

All the relative abundance ions for ERY A compund were below 20%, meeting the criteria.
